# Clinical Recognition of FAS: Difficulties of Detection and Diagnosis

**Published:** 1994

**Authors:** Jon M. Aase

**Affiliations:** Jon M. Aase, M.D., is clinical associate professor of pediatrics at the University of New Mexico, Albuquerque, New Mexico

## Abstract

Verifying the diagnosis of FAS in a specific patient is often difficult. Future diagnostic aids may include weighted checklists, laboratory and imaging studies, and psychological profiles.

Until about 20 years ago, the dangers of maternal alcohol consumption to the developing fetus were generally minimized or dismissed. Reports linking maternal alcohol use and fetal growth deficiency emerged from France in the late 1950’s. Then in 1968, Lemoine and colleagues (1968) described growth deficit, mental retardation, and an unusually high rate of birth defects in 127 children born to alcoholic mothers. In addition, Ulleland (1972) found pre- and postnatal growth deficiency and developmental abnormalities in 8 of 12 children born to alcoholic mothers.

Based in part on these cases, Jones and Smith (1973) introduced the term “fetal alcohol syndrome” (FAS) to describe a pattern of abnormalities found in some children born to alcoholic women. Jones and Smith’s definition was significant because it clearly delineated a clinically recognizable syndrome that was distinct from all other patterns of congenital malformation and that was seen exclusively in the offspring of mothers who drank large amounts^1^ of alcohol during pregnancy.

During the next few years, more than 100 patients with FAS were reported. The earliest descriptions concentrated on the most severely affected patients, in whom the syndrome was most clearly recognizable. It soon became apparent, however, that the diagnosis of FAS could encompass children with varying degrees of growth failure and mental deficiency who did not display all of the physical abnormalities originally considered essential for the diagnosis.

Most birth defect syndromes undergo expansion and redefinition of their diagnostic criteria as more cases are recognized. This refinement is useful for the identification of patients with mild or atypical symptoms. However, confirmation of the diagnosis in atypical cases may become difficult or impossible when the primary diagnostic features are relatively common nonspecific abnormalities or are occasionally found in normal individuals. This is true in FAS, as described in more detail below. Diagnosis of FAS is complicated further because the mechanisms by which alcohol causes fetal damage are still unknown. This article discusses the current criteria for diagnosing FAS.

## Clinical Features of FAS

Jones and coworkers (1973) defined three major categories of abnormality in children with FAS:

- Slow growth both before and after birth, involving height, weight, and head circumference
- Deficient intellectual and social performance and muscular coordination
- A consistent pattern of minor structural anomalies of the face, together with more variable involvement of the limbs and heart.

With some modification, these criteria still form the basis for the clinical diagnosis of FAS. Evidence of abnormality in all three areas is enough to exclude most other birth defect syndromes, but documentation of maternal alcohol use during pregnancy is required for complete confirmation.

### Growth Abnormalities

Children with FAS characteristically grow slowly during childhood, and many of the most severely affected children also experience intrauterine growth retardation. Statural growth and weight gain are suppressed at different rates, however. In classic cases, FAS children grow taller at about 60 percent of the normal rate through early childhood, while their weight increases at about 33 percent of the normal rate. Thus, they appear unusually slender or even malnourished despite an adequate diet.

Slow growth in head circumference indicates slow brain growth, a consistent feature in infants, children, and adolescents with moderate to severe FAS. Small brain size has been confirmed at autopsy, where abnormalities of brain development also are evident. The intellectual and behavioral abnormalities in children with FAS—as well as an increased risk of seizures—are results of the effects of alcohol on the developing brain.

### Physical Abnormalities

The pattern of physical abnormalities in FAS includes subtle abnormalities in the face; limitations of joint movement; and other, less frequent anomalies. Although the overall facial appearance is the single most helpful clue in the diagnosis of FAS, each specific facial abnormality represents a minor anomaly or a variant of normal and thus may escape notice unless specifically looked for by the diagnostician.

**Figure 1:**
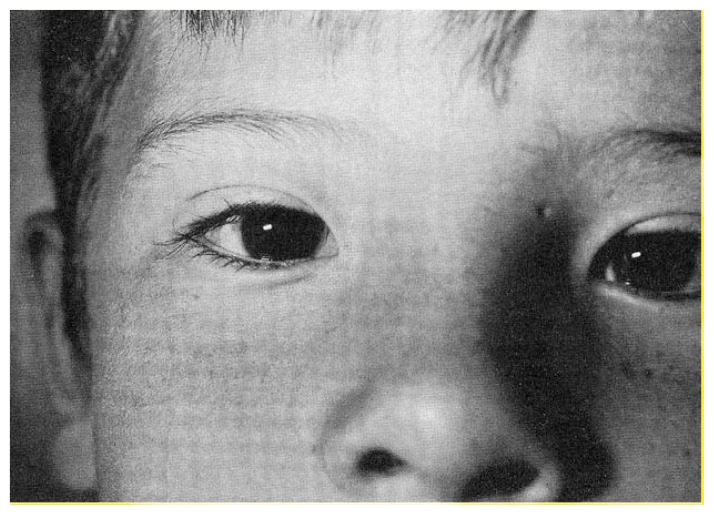
Eyes and midface of a child with FAS, showing short eye openings and drooping eyelids. Photograph courtesy of the author.

One of the most distinctive and consistent signs of FAS is found in the eyelids. Affected children often appear to have widely spaced eyes (figure 1), although measurements reveal that the eyes are normally situated. This discrepancy is caused by short palpebral fissures (eye openings). That is, the distance between the inner and outer corners of each eye is shortened, making the eyes appear smaller and farther apart than normal. Measurement of palpebral fissure length is needed to confirm this observation, because a similar appearance can arise from other causes. Drooping of one or both eyelids also is seen frequently in FAS.

Slow growth in the center of the face is another hallmark of FAS. This slow growth produces a hypoplastic (under-developed) midface; the zone between the eyes and mouth may seem flattened or depressed, and the bridge of the nose is often low. Slow nasal growth in the outward direction, away from the plane of the face, may leave a crescent-shaped fold of skin covering the inner corner of the eye (figure 1). If the nose grows slowly in length, from its attachment between the eyebrows to the tip, the nostrils often point forward as well as downward.

**Figure 2:**
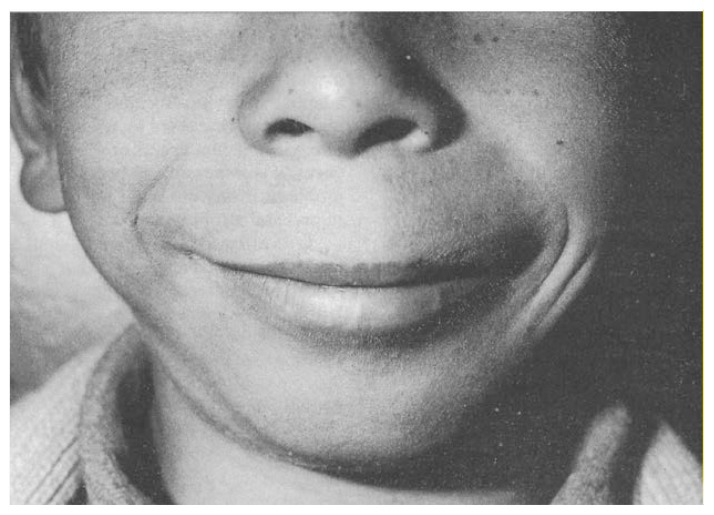
Lower face of a child with FAS, showing slightly short nose, long middle part of the upper lip without a central groove, and narrow red margin of the upper lip. Photograph courtesy of the author.

Another subtle but characteristic facial feature in children with FAS is found in the philtrum, the zone between the nose and the mouth. This region normally is characterized by a vertical midline groove, bordered by two vertical ridges of skin. Where the groove meets the red margin of the upper lip, an indentation usually is seen, producing a “cupid’s bow” configuration of the lip. The classic FAS face shows a long, smooth philtrum without ridges and with a smoothly arched upper lip margin (figure 2).

**Figure 3:**
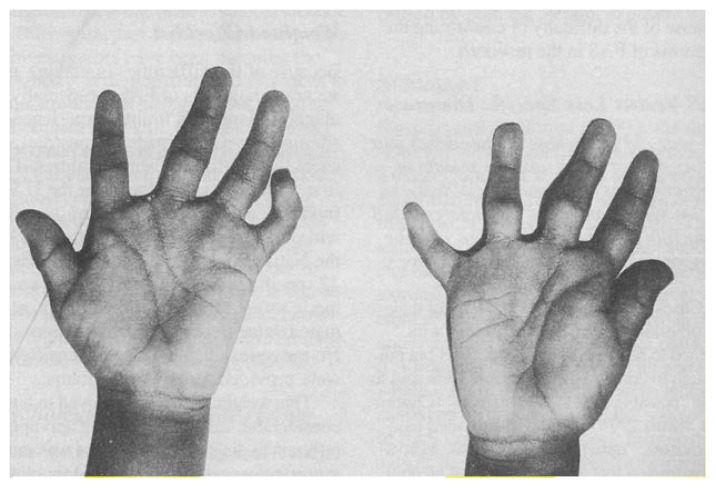
Hands of a child with FAS, showing abnormal finger joints, curved fingers, and longitudinal palmar creases. Photograph courtesy of the author.

Abnormalities of limbs and joints are less consistent features but are seen much more frequently in children with FAS than in the general population. For diagnostic purposes, the most useful such characteristics include deformities of the small joints of the hands as well as incomplete rotation at the elbow. In the hands, the typical anomalies include longitudinally oriented palmar creases; an inability to straighten a finger at one or more joints; and curving of a finger sideways, toward the middle finger (figure 3).

Children with FAS also are at increased risk for many common birth defects that are not necessarily associated with alcohol. Congenital heart disease, cleft lip and palate, anomalies of the urinary tract and genitals, and spina bifida (a defect of the spinal column) occur 5 to 60 times more often in children who were prenatally exposed to alcohol than in the general population (Abel 1990). Because none of these abnormalities is specific to FAS, they can only help to support, but not to define, the diagnosis.

### Intellectual and Behavioral Abnormalities

The first reports of FAS documented delayed intellectual and behavioral development in all children studied; however, all of these children were younger than school age. Further study revealed a variety of learning and behavior problems that appeared at different ages. In infancy, feeding problems, irritability, and unpredictable patterns of sleeping and eating make the babies hard to care for and interfere with maternal bonding. During the preschool years, the children are described as affectionate but very active, “flighty” and “distractible” (Streissguth 1986). Poor fine motor coordination also becomes apparent at this age.

In the first few years of school, most children with FAS receive the diagnosis of attention-deficit hyperactivity disorder (ADHD)^2^ because of their high activity level, short attention span, and poor short-term memory (Landesman-Dwyer et al. 1981). Many need special educational help, even if their IQ falls within the normal range.

As the FAS children grow older, their school progress is further compromised by inadequate communication skills, impulsivity, and difficulty with social interactions. Their adolescence is marked by poor judgment, trouble with abstract thinking, and limited problem-solving skills. Many affected children eventually drop out of school, and their ensuing integration into society is tenuous at best (Streissguth et al. 1991) (see the article by Streissguth, pp. 74–81).

In the few studies of long-term social outcome that currently exist, anecdotal reports and case histories of adolescents and adults with FAS show a distressingly similar pattern (Dorris 1989). They have difficulty finding and holding jobs because of their unreliability; lack of social skills; and often, functional illiteracy. Establishing and maintaining lasting interpersonal relationships also is a common problem. For this reason, affected adults often lack a social support system and therefore have a higher-than-average risk of becoming involved in drug abuse and criminal behavior.

## Diagnostic Pitfalls

In summary, the current criteria for the diagnosis of FAS depend on recognition of a consistent pattern of minor, often subtle physical anomalies; generalized but disproportionate growth retardation; and nonspecific developmental and behavioral aberration. Some of these characteristics change with time, and their degree of severity may vary among individuals. Confirming the diagnosis of FAS in a specific patient is often difficult, even for clinicians with considerable experience with FAS.

Underdiagnosis usually occurs when the complete pattern of abnormalities cannot be substantiated, often because of the patient’s age, racial background, or familial characteristics. Clinicians also may be reluctant to make the diagnosis because they fear stigmatizing both the mother and the child (Morse et al. 1992). Overdiagnosis may result from too much emphasis on maternal drinking history, the presence of nonspecific abnormalities, or failure to recognize a different but similar congenital disorder. These problems are discussed below.

### Changes in Features With Age

Recently, it has become clear that many of the critical diagnostic features of FAS change as the child grows older. The diagnosis is most difficult in newborns and adults. For example, relative mid-facial hypoplasia and a short nose with a low nasal bridge are normal features of many babies in the newborn period; however, if these features persist into the second year of life and beyond, they may be diagnostically helpful. Conversely, continued slow growth of the face and nose through adolescence compensates for earlier midfacial hypoplasia and obscures the typical facial appearance of individuals with FAS as they pass into adult life.

New information also suggests that prenatal growth retardation is found in only about 70 to 75 percent of children eventually diagnosed as having full-fledged FAS (Hymbaugh et al. 1993). The characteristic slender body build begins to change during adolescence in many affected children, especially girls, who may become moderately obese in late adolescence (Streissguth et al. 1991). Final adult height and head circumference, however, tend to remain below normal and thus have some diagnostic value at later ages.

Judgments of intellectual performance and behavior are critically dependent on the patient’s age. As mentioned above, the typical learning problems and mal-adaptive behavior in children with FAS continue to evolve throughout childhood and into adolescence, involving far more than simple mental retardation. Therefore, any psychologically based diagnostic criteria must take into account the patient’s developmental stage. For example, testing for elementary school-aged children might concentrate on measures of attention and memory, whereas that for teenagers might focus on judgment and self-regulation.

### Influence of Racial and Familial Traits

Because most of the clinical features of FAS are not discrete abnormalities but fall somewhere along a continuum, it is important to consider the normal variation of features in the patient’s racial group or family. For example, a moderate degree of midfacial hypoplasia is a normal characteristic in many Native American groups and should be considered when examining children from that population. Similarly, the broader lips normally seen in children of black parentage may cancel out the narrowing of the upper lip border in children with FAS. Also, the tall stature of some northern European and central African populations may compensate to a large degree for the statural growth deficiency of a child with FAS.

Similar modification of the classic FAS characteristics may be related to inheritance within a particular family. Two parents with very high IQ’s may have a child with FAS whose intelligence is within the normal range but well below what would be expected, based on the parents’ intellect. Height growth, some facial features, and even creases on the palms may be influenced so much by heredity that the signs of FAS are obscured or mimicked.

### Similarities to Other Disorders

Few birth defect syndromes resemble FAS, and they can be distinguished on examination. Nevertheless, children with a wide variety of other diagnoses have been initially suspected of having FAS. Such errors usually result when only a few features are considered, while the broader pattern goes unnoticed. Thus, a child with Cornelia de Lange syndrome (a hereditary form of mental retardation) may have slow growth, a small head, and a long philtrum, but the overall constellation of features is quite different from that of FAS. Often, a physician must correct an erroneous diagnosis of FAS given to a child who has some other disorder with vastly different implications.

### Nonspecific Abnormalities

Most of the difficulties with diagnosis described above are based upon one underlying theme: none of the abnormalities found in FAS is specific to that diagnosis. Many otherwise normal children may display one or two of the common features of FAS. Isolated mental deficits or behavioral abnormalities also may occur as part of birth defect syndromes other than FAS. Slow statural growth, for example, occurs in more than 600 conditions, and small head circumference is found in about 250. Mental deficiency is a recognized component of some 700 physical deformity syndromes; it also occurs as an isolated feature in 2 to 4 percent of most populations in the absence of any abnormal physical features or history of prenatal exposure of any kind. Beyond this, some of the behavioral manifestations so frequently displayed by children with FAS can be part of a broader pattern that has recently been associated with abuse and neglect in early infancy and lack of parental bonding (Magid and McKelvey 1987).

### Distribution in Various Populations

Many of the earliest case reports and epidemiologic studies of FAS involved Native American subjects, giving rise to the mistaken impression that FAS is peculiar to Native American populations. This misconception may have led to overdiagnosis of FAS in Native American groups and perhaps to underdiagnosis in other groups. In fact, FAS has been found in every population that uses alcohol.

**Figure 4:**
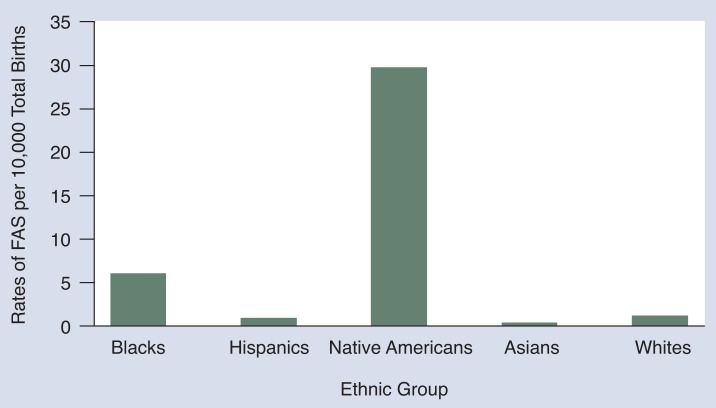
Relative frequency of FAS in newborns in the United States by ethnic group, 1981–1986. SOURCE: Adapted from Chávez et al. 1988.

In the United States, epidemiologic data suggest that the rates of FAS tend to be higher in blacks and Native Americans than in whites of similar socioeconomic status and areas of residence (Abel 1990). A survey compiled by the Centers for Disease Control and Prevention (CDC) (Chávez et al. 1988) reviewed more than 4.6 million births in approximately 1,200 U.S. hospitals and showed considerable differences in occurrence of FAS among racial subgroups (figure 4). The reasons for the variance among these groups remain unclear, but the figures must be interpreted with caution because of the difficulty of confirming the diagnosis of FAS in the newborn.

### FAS Versus Less Specific Diagnoses

A 3-year-old girl, whose mother drank four bottles of beer a day until the middle of pregnancy, has no unusual facial features, but her weight and head circumference fall at the fifth percentile for her age, and she is hyperactive with a short attention span.

Cases such as this provide a major diagnostic dilemma. Initially, children who seemed to have several signs of FAS but not enough to confirm the diagnosis, were said to have “possible fetal alcohol effects” (Clarren and Smith 1978). This was intended as a “bookmark” rather than a diagnosis, indicating suspicion that although prenatal alcohol exposure could be responsible for the abnormalities, further proof was needed to confirm the diagnosis. Unfortunately, the word “possible” was soon dropped, and fetal alcohol effects took on a life of its own as a distinct diagnosis. Often, this judgment was based primarily on acknowledged or suspected maternal alcohol abuse during pregnancy.

In one epidemiologic study (May et al. 1983), only about one-third of babies born to mothers who drank heavily during pregnancy were found to have classic FAS, whereas almost one-half seemed to be entirely normal. Roughly 20 percent of the children were not categorized as having FAS but had some combination of mental deficit, growth delay, and mal-adaptive behavior. Because these abnormalities can be caused by a wide variety of environmental agents or genetic influences, it is impossible to prove that the problems in any one child were the result of prenatal alcohol exposure.

In an effort to clarify the situation, other terms were introduced, such as “alcohol-related birth defects” (Sokol and Clarren 1989). These concepts may be helpful in the epidemiologic study of large groups of children exposed prenatally to alcohol, but they have little use in the identification of FAS in a specific individual. Until a precise and unequivocal standard for diagnosis becomes available, the wisest course would be to provide a purely descriptive diagnosis for children who do not meet the criteria for FAS. Thus, the child described above could be categorized simply as alcohol exposed in utero, deficient in weight and head circumference, and at risk for ADHD.

## Aids to Diagnosis

### Weighted Checklist

Because of the difficulties in making an accurate diagnosis of FAS—especially for diagnosticians with limited experience—attempts have been made to construct checklists of distinguishing features. The first such list was produced for the U.S. Indian Health Service as part of its nationwide effort to define the extent of FAS in the Native American population. Each of 42 specific features was assigned a numeric score, or weight, based on its estimated value in confirming the diagnosis. No measures of specificity or sensitivity were provided for any of the items.

This weighted checklist proved to have considerable value in increasing appropriate referrals to diagnostic clinics but was much less effective as a diagnostic tool for clinical purposes. A newer, shorter, and more scientifically based checklist is being developed by the author and the CDC and should be ready for testing sometime in 1994.

### Laboratory and Imaging Studies

No laboratory tests are available that can establish or rule out the diagnosis of FAS. However, a growing research effort is directed toward finding the underlying mechanisms that contribute to fetal alcohol damage. Scientists also are searching for genetic and biochemical characteristics associated with susceptibility to FAS. Both of these lines of research hold promise for eventual development of tests to show which mothers, babies, or pregnancies are at highest risk for adverse effects of alcohol.

During the past 3 years, several groups of investigators have been pursuing the possibility of demonstrating differences in brain structure by using special imaging techniques. Magnetic resonance imaging (MRI) and positron emission tomography (PET) already have proved useful in the diagnosis of other disorders involving brain dysfunction. MRI uses a magnetic field to produce an image of the living brain; PET uses radioactive isotopes to monitor brain metabolism. There is considerable interest in applying these technologies to the study of children with FAS (see the article by Mattson et al., pp. 49–52).

### Psychological/Behavioral Testing

As discussed earlier, the physical features of FAS are nonspecific, whereas their overall pattern is unique. Similarly, the pattern of learning and behavioral characteristics may have diagnostic value in affected children. The brain is the organ most sensitive to the prenatal damage caused by alcohol. If that damage resulted in a unique, unmistakable psychological profile, FAS could be diagnosed reliably without reliance on the variable and subtle clinical features. As yet, no such distinctive profile has been ascertained, and reliable measures of some of its components remain to be developed. If controls can be introduced for such nonspecific confounding factors as physical or emotional abuse and neglect, psychological-behavioral assessment may prove to be the long-sought “gold standard” for diagnosis of injury caused by prenatal alcohol exposure.

## Summary

Since its first definition in 1973, the diagnosis of FAS has relied on the recognition of a unique pattern of structural and functional abnormalities in children with a history of exposure to alcohol before birth. The individual abnormalities are often subtle and hard to detect and mostly represent the extreme of a normal spectrum of development. For these reasons, FAS is both under-and overdiagnosed. In individual patients, it is impossible to determine with certainty whether their abnormalities were indeed caused by alcohol unless the full spectrum of FAS symptoms is present. Diagnostic aids in the form of checklists, laboratory and imaging studies, and psychological profiles may be of some assistance, but the definitive confirmatory test for intrauterine alcohol damage has not yet been developed.

## Footnotes

1 FAS researchers do not always specify the amount of maternal drinking involved. Nonspecific phrases such as “heavy drinking” used in this article reflect the terminology of the reference cited.

2 Attention-deficit hyperactivity disorder (ADHD) is a disorder of childhood characterized in general by levels of restlessness and distractibility that impair school and social functioning.
